# What If the Clinical and Older Adults’ Perspectives about Frailty Converge? A Call for a Mixed Conceptual Model of Frailty: A Traditional Literature Review

**DOI:** 10.3390/healthcare11243174

**Published:** 2023-12-15

**Authors:** Asya Hani Khalil, Robbert J. J. Gobbens

**Affiliations:** 1 Hariri School of Nursing, American University of Beirut, Beirut 1107 2020, Lebanon; 2 Faculty of Health, Sports and Social Work, Inholland University of Applied Sciences, 1081 HV Amsterdam, The Netherlands; robbert.gobbens@inholland.nl; 3 Zonnehuisgroep Amstelland, 1186 AA Amstelveen, The Netherlands; 4 Department of Family Medicine and Population Health, Faculty of Medicine and Health Sciences, University of Antwerp, 2610 Wilrijk, Belgium; 5 Tranzo, Tilburg University, 5037 DB Tilburg, The Netherlands

**Keywords:** frailty, clinical, older adult perspective, perception, lived experience, mixed conceptual model

## Abstract

Existing frailty models have enhanced research and practice; however, none of the models accounts for the perspective of older adults upon defining and operationalizing frailty. We aim to propose a mixed conceptual model that builds on the integral model while accounting for older adults’ perceptions and lived experiences of frailty. We conducted a traditional literature review to address frailty attributes, risk factors, consequences, perceptions, and lived experiences of older adults with frailty. Frailty attributes are vulnerability/susceptibility, aging, dynamic, complex, physical, psychological, and social. Frailty perceptions and lived experience themes/subthemes are refusing frailty labeling, being labeled “by others” as compared to “self-labeling”, from the perception of being frail towards acting as being frail, positive self-image, skepticism about frailty screening, communicating the term “frail”, and negative and positive impacts and experiences of frailty. Frailty risk factors are classified into socio-demographic, biological, physical, psychological/cognitive, behavioral, and situational/environmental factors. The consequences of frailty affect the individual, the caregiver/family, the healthcare sector, and society. The mixed conceptual model of frailty consists of interacting risk factors, interacting attributes surrounded by the older adult’s perception and lived experience, and interacting consequences at multiple levels. The mixed conceptual model provides a lens to qualify frailty in addition to quantifying it.

## 1. Introduction

As a geriatric complex condition that could render the older adult vulnerable to multiple adverse health outcomes, frailty has gained the attention of scholars worldwide over the past three decades. Despite the advances in frailty research, consensus on its definition is still lacking, and how the findings of such frailty research can be applied in practice and naturally occurring settings is still questionable [1]. The prevalence of frailty varies across studies depending on what frailty definition and, subsequently, what frailty measurement tool was adopted. One meta-analysis investigated the prevalence of frailty, which was 26.8% among multiple settings and contexts, and showed that one out of four older adults is frail, and one out of two older adults in nursing homes is frail [2].

Three major frailty models appear in the literature: the frailty phenotype model by Fried et al., the cumulative deficit model by Mitnitski and Rockwood, and the integral conceptual model by Gobbens et al. The first two models define frailty as a syndrome of decreased physiological capacity among multi-systems, resulting in impaired function of the older adult upon exposure to even minimal environmental challenges leading to disability [3]. The frailty phenotype model views frailty from a biomedical biological basis, focusing solely on physical aspects [4]. The cumulative deficit model views frailty as a multidimensional concept resulting from deficit accumulation that increases with age, among which are psychological aspects [5,6]. The integral conceptual model views frailty from a bio–psycho–social perspective and defines frailty as a dynamic state that influences the person who lives the experiences of losing in one or more domains of human functioning (whether physical, psychological, or social) [7]. In Gobbens’ definition, these losses are the result of a range of variables and lead to an increased risk of adverse outcomes [7]. The three models are widely used in research and practice, with each giving rise to one or more frailty measurement tool(s). However, none of the mentioned models accounted for the perspective of older adults themselves when defining frailty and operationalizing it. The perspective of older adults would be less significant if frailty were a biological/pathological disease, restricting its definition to the medical field. But frailty is not a disease, or there would be an agreement on one uniform definition. Also, there would not be arguments for psychological and social frailty attributes. Qualitative studies exploring the perceptions of frail older adults about frailty are scarce. A scoping review found 10 studies exploring the perceptions of frailty among community older adults up to 2019, 8 of which are primary studies and conducted in Western countries [8]. The conflict emerged when those few qualitative studies conducted on perceptions of frailty, showed that older adults refuse to be labeled as “frail” even if we considered them “clinically frail” by means of frailty measurement tools [8,9,10,11,12]. Thus, highlighting the importance of considering the perspective of older adults about frailty.

Shifting the lens from a purely evaluative one to an interpretive one is worthy of minimizing the gap between the clinical perspective of hasty frailty screening and the older adults’ perspective of refusing the frailty labeling. To resolve controversies around frailty conceptualization, focusing on how older adults perceive and experience frailty is a future must. When healthcare professionals, including nurses, understand the perceptions and lived experiences of older adults with frailty, they will be able to adopt communication strategies and interventions accordingly. Thus, we aimed to propose a mixed conceptual model of frailty that builds on the integral conceptual model [7] while accounting for older adults’ perspectives and lived experiences of frailty.

## 2. Methods

### 2.1. Information Sources

We conducted a traditional literature review using the journal database of PubMed and Google Scholar accessed through the AUB online library. Since the development of a model necessitates the exploration of several aspects of frailty, the PubMed database supports conducting a comprehensive search. PubMed contains peer-reviewed journal articles of different study designs that originate from both biomedical and behavioral sciences. To broaden our search, we used Google Scholar for possible additional studies from the grey literature. We included further relevant papers by ancestry method through using the reference list of selected articles.

### 2.2. Literature Search

For the literature search, we did not impose a specific study design or date limits. We excluded articles that targeted frailty in a specific population with a certain pathology (secondary frailty) and those where frailty was studied in a population below 60 years of age. We also excluded papers available in languages other than English, those where frailty was not mentioned as the main concept, and those studying frailty in animals. We included a total of 73 articles to develop the mixed conceptual model of frailty. We used the following key terms: frailty, frail, “older adults”, elderly, defin*, model, attribute, phenotype, “concept analysis”, meaning, theor*, perception, experience, “lived experience”, screening, tool*, identif*, risk factor*, cause*, etiology, antecedents, determinant*, histor*, consequence*, effect*, outcome*, impact, result*. For concept-related terms, we utilized the Boolean operator “OR”, and for combining several concepts, we used the Boolean operator “AND”.

### 2.3. Data Extraction

We scanned the titles, abstracts, and full texts to see if the content matched the following data to be extracted: frailty definition, whether theoretically or operationally; frailty risk factors; frailty consequences; frailty perceptions; and lived experience from older adults’ perspectives.

### 2.4. Data Synthesis

Synthesis of findings was tabular for frailty risk factors and narrative for all other findings. Themes and subthemes emerging from qualitative data were those common in meaning among more than one study and those that showed up by means of inductive thematic analysis (with no prior framework for analysis) [13].

## 3. Results

### 3.1. Defining Attributes of Frailty

Despite the absence of a gold standard definition of frailty and accounting for the different models of frailty, the following defining attributes were the most common ones appearing in the literature up to date:

Vulnerability/susceptibility, aging, dynamic, complex, physical, psychological, social.

*Vulnerability/susceptibility* appears in most of the cited definitions of frailty [14,15,16]. However, vulnerability is not a synonym for frailty since vulnerability can be static rather than dynamic [15]. All frail older adults are vulnerable, but not all vulnerable older adults are frail [15]. Vulnerability means the probability of being influenced by small changes (biomedical, psychosocial, or environmental) [14], which could be internal fragile health due to deficiency in internal capacities and/or external through exposure to harmful factors that impact one’s capabilities [14].

*Aging* and its association with frailty is not fully understood. There is an overall agreement that aging is a critical component of frailty [16], but not all older adults are frail [14]. In the three major models mentioned above, frailty was studied among the population of older adults, and subsequently, older adults were the ones referred to within the definitions of frailty.

The *dynamic* state of frailty is a defining attribute explicitly stated in the integral conceptual model of frailty [7]. That means there is a fluctuation between good health times and bad health times for frail older adults, and as frailty progresses, symptoms are more apparent [14]. These symptoms may be of sudden onset if a trigger, such as infection or injury, or even psychosocial stressors, occur, resulting in sudden immobility or delirium [15,16]. Transitions between frailty states are also possibly referred to as a “tenuous nature of frailty”, putting frailty on a continuum of being less or more frail and as a midpoint between independence/robustness and pre-death/disability [15]. This dynamic continuum supports the idea that frailty is preventable, reversible, and can be delayed or palliated, considering the severity and the influence of frailty [14,17].

Referring to frailty as a *complex* phenomenon is mostly found in frailty definitions as “multifactorial”, “multidimensional”, and “across multiple systems” [15,16,18,19]. What makes such a “multidimensional” feature complex is the interrelation across systems (physical, psychological, social) in frailty. That is because such interrelation occurs with synergy at multiple levels: at the level of frailty determinants/risk factors, at the level of its clinical manifestations, and at the level of its trajectory and consequences [15,16]. Synergy at the level of frailty determinants means that the additive effects of different types of frailty risk factors may be greater than their sum, and the contribution of these factors could result in worsening frailty status. Synergy at the level of clinical manifestations appears when physical, social, and psychological aspects interact, and mild physiological and psychosocial stressors increase the likelihood of adverse outcomes in frail older adults [15,16]. At the consequences level, frailty consequences are interactive on different levels; for example, frailty worsens comorbidities, which in turn can worsen to a disability, probably ending by institutionalization and family financial strain [15]. Thus, *complex* as a frailty attribute is in accordance with the complex system meaning in the complex systems theory. A complex system, in the complex systems theory, consists of a large number of parts interacting in a pragmatic way [20]. In such systems, the entire system is more than the summation of its parts; given the characteristics of its parts and the laws of their interactions, one could infer the properties of the whole system [20]. Fried and colleagues, in a recent appealing paper, supported the use of complex systems theory when studying physiological frailty [21].

*Physical* in the context of physical frailty is an agreed-upon feature of frailty. Sometimes referred to as physiological, biological, phenotype, or physical function component [14,18]. Physical signs and symptoms/clinical manifestations of frailty are as follows: changes in anthropometric measurements; sarcopenic obesity; malnutrition; decrease in the level of physical activity; decrease in hand grip strength; exhaustion; unintentional weight loss; changes in gait; changes in cardiac autonomic modulation; poor sleep quality; comorbidities; anorexia; increase in estradiol levels; increase in levels of plasma proteins, such as adiponectin and inflammatory glycoproteins; and use of auxiliary devices [22].

*Psychological* in the context of psychological frailty is common in definitions that follow mainly the integral conceptual model and the cumulative deficit model [5,6,7]. In some papers, psychological frailty refers to a decline in motivation and positive mood [14]. In others, psychological frailty is an umbrella term that includes cognition, mood, and motivation [23]. Cognitive frailty as a term is new and is little cited in the literature as compared to physical frailty, but it is parallel to physical frailty in the loss of resilience and adaptability in the aspect of brain function and implies a linkage to physical frailty [23]. In 2013, the International Academy on Nutrition and Aging (I.A.N.A) and the International Association of Gerontology and Geriatrics (I.A.G.G) first defined cognitive frailty. In their definition, cognitive frailty clinically manifests as the presence of both physical frailty and mild cognitive impairment simultaneously while ruling out concurrent Alzheimer’s dementia or other types of dementia [24]. Within the psychological frailty umbrella, there is mood and motivational frailty. Mood is a relatively constant condition of emotion, such as anxiety, depression, fear, or anger [23]. Motivation means a desire toward a goal or a lack of that desire (apathy), but mood and motivation can be independent [23].

As with psychological frailty, *social*, in the context of social frailty, is poorly studied in the literature as a solitary concept and mainly found in the integral definitions of frailty [25,26]. One suggested definition is that social frailty is a continuum from being at risk of losing to having lost resources that are important for fulfilling one or more basic social needs during the life span [25]. These resources, per this definition, can be social resources such as children or spouses, social behaviors and activities such as social participation, and self-management abilities such as feeling empowered and autonomous in making decisions [25]. Some studies revealed a distinct association between social vulnerability and overall frailty [25]. Furthermore, several studies found a reciprocal association between social frailty and physical frailty [26].

### 3.2. Perceptions and Lived Experiences of Frailty in Older Adults

Perceptions and lived experiences are unique to every individual; however, we list some of what our search revealed in terms of themes/subthemes related to frailty perceptions and lived experiences by older adults.

#### 3.2.1. Refusing Frailty Labeling Explicitly [8,9,10,11,12] and Additional Labeling Subthemes

*Being labeled as frail “By Others”, as compared to “Self-Labeling”:* Labeling clinically frail older adults as frail “by others”, as compared to “self-labeling”, reflected in confirming a frailty identity for these older adults [27].

*Frailty identity demotivated older adults:* The frailty identity demotivated older adults from engaging in physical and social activities, negatively affected their physical health, and provoked further stigmatization [27].

*From the perception of being frail towards acting as being frail:* The perception of being frail resulted in older adults acting as being frail and was related to losing dignity, control, and independence [8].

*Positive self-image:* Some frail older adults viewed themselves as resilient, independent, and autonomous despite physical frailty [9]. Some “clinically frail” older adults did not perceive themselves as frail and had a good quality of life [28].

*Skepticism about frailty screening:* Some older adults had skepticism about what actions could be performed after the screening, particularly when the health resources are insufficient [29].

*Communicating the term “Frail”:* There was a concern related to communicating the term “frailty” while screening for it, as this labeling carried stigmatization, hindered participation in positive health practices, and deprived the older adult of the choice of being frail or not [29].

#### 3.2.2. Negative Impacts and Experiences of Frailty

▪ *Incapable body:* Older adults experienced physical limitations and an incapable body, sometimes referred to as the shutting down of the body [30,31].
▪ *Unpredictable body:* The bodies of the older adults living with frailty predicted their daily schedule; alterations and fluctuations in the body’s capacity each day determined the older adult’s daily activities [30,31].
▪ *Synergy between low mood and incapable body:* When older adults with incapable bodies experienced low mood, their physical limitations intensified [31].
▪ *Anxiety and fear:* Older adults were worried about how risky their usual activities might become when they have changes in their bodies [31]. Fear also existed surrounding falling and hurting themselves when performing physical exercises [32].
▪ *Dependency in daily activities:* Some older adults living with frailty were dependent on their family members or requested the continuous help of professionals [32].
▪ *Shrinkage in social network:* Older adults living with frailty experienced a gradual decrease in their in-person contact with others, especially on days when their bodies had functional limitations [32].

#### 3.2.3. Positive Impacts and Experiences of Frailty

▪ *“Not giving up” and coping:* Frail older adults knew their bodies more deeply and tried to adjust their usual activities or consider alternative ones to maintain independence in activities that are worthy for them [30,31,32].
▪ *Rebuilding, fighting, and keeping going:* Older adults with frailty and multimorbidity maximized their quality of life through rebuilding and fighting to maintain social relationships and engaging in self-care [33]. Rebuilding refers to how older adults rearrange their lifestyles to reach stability and how they use gratitude and positive life attitudes to enhance their psychological prosperity [31,33].
▪ *Recognizing the value of being active:* Older adults living with frailty were motivated to perform physical exercises, some preferring to do it within a group and others at home [32]. Mental activities such as crosswords and reading were considered by older adults as a form of being active [32].

### 3.3. Frailty Risk Factors

We present here a synthesis of frailty risk/associated factors from combined sources reflecting what we know so far. Interestingly, some risk factors serve as attributes or consequences of frailty in other papers. This would reflect two possibilities: either the association between frailty attributes, antecedents, and consequences is bidirectional with possible interactions at all potential levels supporting the complex feature of frailty explained earlier, or there is a discrepancy in conceptualizing frailty across scholars due to different philosophical and theoretical perspectives. Synthesis of frailty risk/ associated factors yielded the following classification (Table 1):

### 3.4. Frailty Consequences

Most of the studies conducted on frailty outcomes showed that frailty is associated with negative adverse outcomes independent of comorbidities [53]. A meta-analysis showed that frailty is linked approximately to 2.3 times the risk of premature mortality, 2 times the risk of disability (decline in activities of daily living), 1.8 times the risk of hospitalization, up to 2.6 times the risk of physical limitation, and up to 2.8 times the risk of falls and fractures [54]. Another systematic review and meta-analysis found that decreased cognitive function is a clinical outcome of frailty, and cognitive performance declines as the older adult moves from pre-frailty to frailty [55]. Frailty can complicate medical and surgical conditions, lead to fear of falling, worsen the quality of life of older adults [22], and predict institutionalization [56]. In addition, frailty has been shown to be a predictor of overall mortality by more than five times in older adults infected with COVID-19 [57]. Frailty negative outcomes are not just limited to the individual level but extend to the caregiver/family, healthcare sectors, and societal levels. For instance, frailty was associated with caregiver burden and financial strain [15,58]. Greater utilization of social care resources is required for older adults living with frailty [59]. Independent of aging and comorbidities, severe physical frailty was associated with more than 30 times increases in health care costs as compared to mild pre-frailty status [60]. Also, frailty predicted increased admission to intensive care units, invasive mechanical ventilation, and increased hospital length of stay in older adults infected with COVID-19 [61].

## 4. Discussion

### The Mixed Conceptual Model of Frailty

**Figure 1:**
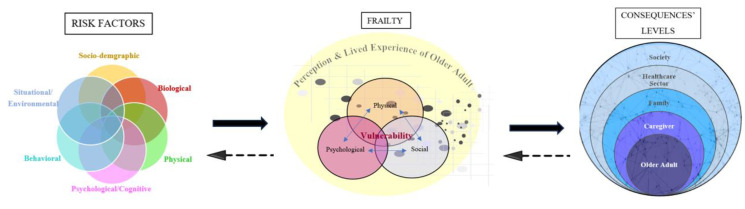
Mixed conceptual model of frailty in older adults.

We present the mixed conceptual model of frailty based on what we previously presented about the state of frailty in the scientific literature in terms of its defining attributes, older adults’ perspectives, risk factors, and consequences. First, we illustrate the mixed conceptual model, and then we break it down into parts to explain the proposed prepositions. Below is the proposed mixed conceptual model addressing frailty in older adults (Figure 1).

**Figure 2:**
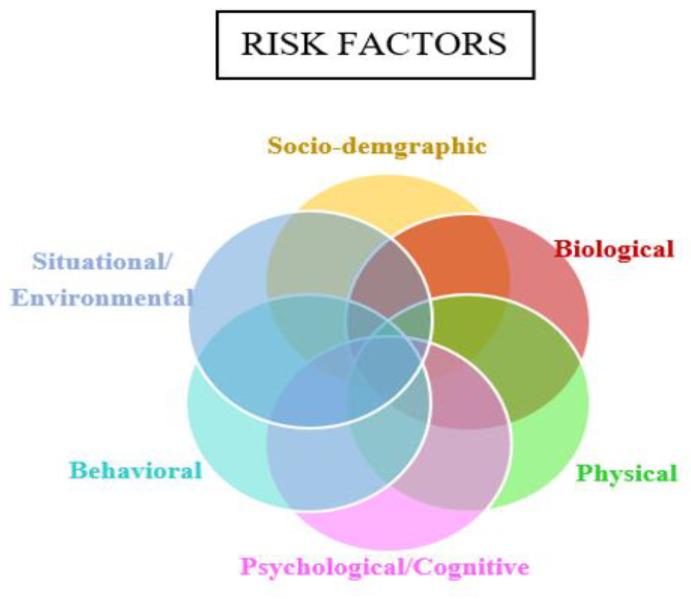
Proposed Frailty Risk Factors Illustration.

For frailty risk factors (Figure 2), the Venn diagram of interconnected circles reflects the synergistic interaction across different frailty risk factors [15,16]: socio-demographic, biological, physical, psychological/cognitive, behavioral, and situational/environmental factors. Dependence across several risk factors leading to frailty is supported in the literature in contrast to a single risk factor causing frailty at a time [35].

**Figure 3:**
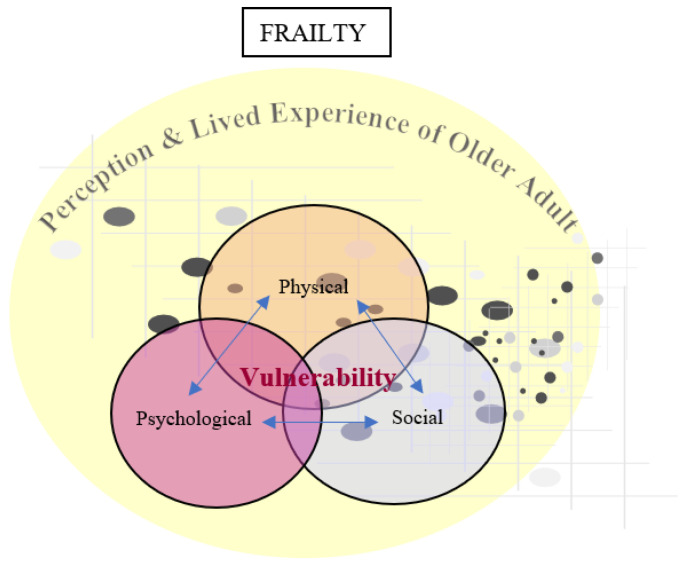
Proposed frailty illustration.

For frailty as a concept (Figure 3), we can classify the defining attributes that we previously presented into frailty content, frailty trajectory, and frailty co-existence. Accordingly, “physical”, “psychological”, and “social” attributes refer to the content of frailty, in addition to the perceptions and lived experiences of frailty. “Complex” and “dynamic” refer to the trajectory of frailty. “Vulnerability” and “aging” refer to the co-existing concepts of frailty.

For the frailty content, there is a common understanding of the multidimensional nature of frailty even in the absence of a gold standard definition [22]. Several scholars in their recent publications are referring to frailty as “physical frailty” when using the frailty phenotype model. Among these scholars is Dr. Linda Fried, the main developer of the frailty phenotype [21], which means physical frailty is part of overall frailty. Other parts/dimensions of overall frailty are psychological frailty, which includes cognition, mood, and motivation [23], and social frailty [7,25,26]. The integral conceptual model is the best model that matches this multidimensionality. According to the integral model, physical frailty, psychological frailty, and social frailty are bidirectionally related, with each could be caused or could lead to the other [7]. We reflected these bidirectional relationships across the frailty dimensions in the blue bidirectional arrows. However, what we added is the perception and lived experience of the older adult with frailty surrounding all frailty dimensions. That is important to account for, as stated previously, because perceptions and lived experiences of older adults would affect frailty prevention, screening, management, and interventions [27]. Also, to study primary frailty, it is reasonable to approach the older adult holistically. Such a holistic approach may inform about other aspects valuable for older living with frailty not considered within the three dimensions (physical, psychological, social). Examples of these could be spiritual and emotional aspects [14]. Such aspects might affect the individual’s coping, adherence, and compliance with management plans.

For the frailty trajectory, the use of the Venn diagram reflects the synergistic interrelation across the physical, psychological, and social dimensions of frailty, supporting the “complex” attribute of frailty. The grey and black dots in the background appearing as a scatter plot reflect the fluctuation in frailty status between more and less frail times, supporting the “dynamic” attribute of frailty [7]. Thus, researchers may consider an X axis and a Y axis in the scatter plot; the Y axis presents frailty severity level as a dependent variable, and the *X* axis presents independent variables yet to be discovered affecting the fluctuation of frailty levels. This illustration comes in accordance with some studies on the factors affecting the fluctuation in frailty status [62,63,64,65,66,67]. Subsequently, possible patterns could be discovered, and perhaps machine learning is an appropriate way to model and initiate that [68,69,70]. Again, such fluctuation would be within the older adult’s perception and experience, as these could differ across frailty severity.

For the co-existing concepts with frailty, we reflected “aging” by adding the word “older adult” to the perceptions and lived experiences of frailty, which is in accordance with illustrating primary frailty in this paper rather than secondary frailty (caused by a certain disease) [71]. We wrote “vulnerability” in red at the center of the three frailty dimensions (physical, psychological, and social). Such a position of “vulnerability” reflects the possibility of it being present in one or more dimensions of frailty [14,25]. However, such vulnerability is again within the perception and lived experience of “clinically frail” older adults, given that vulnerability could imply a negative connotation.

**Figure 4:**
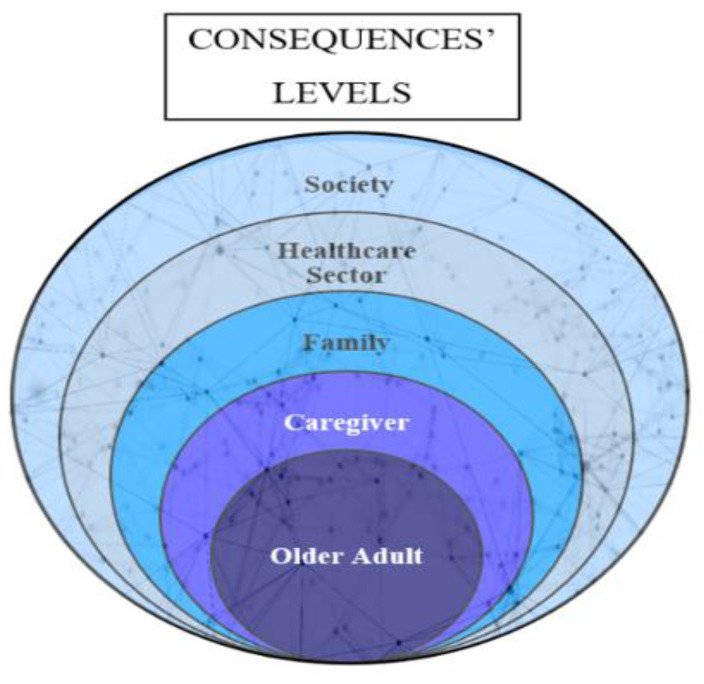
Illustration of proposed frailty consequences levels.

For frailty consequences, these happen at different levels (Figure 4). Frailty affects the individual, the caregiver, the family, the healthcare sector, and the wider community/society, as we previously presented. We utilized the stacked Venn diagram to illustrate these levels from the narrower to the wider level. We could not list all consequences of frailty cited in the literature in this diagram because some consequences can be classified under more than one level, such as institutionalization, which could be under the individual level and under the healthcare sector level. Also, consequences interact synergistically, not always in a predictable way. This supports the complexity of the frailty trajectory and its consequences, where mild physiological and psychosocial stressors increase the likelihood of adverse outcomes in frail older adults [15,16]. Interaction of consequences happens within and between levels. This diagram allowed us to show the overlapping and interconnectedness between these levels and within each level, appearing as complex background networks across and within levels. An example of the interaction across frailty consequences could be falls, fractures, fear of falling, and physical limitation. Within the individual level, falls can lead to fractures, and fractures can lead to physical limitation; falls can also lead to fear of falling [72], and fear of falling can lead to physical limitation [73]. Fractures with fear of falling might prolong physical limitation. An example of a between-levels interaction of consequences might be the surgical and medical complications of frailty at the individual level. Such complications necessitate more hospital visits and subsequently increase costs for the family, increase hospital length of stay, and utilize more social resources afterward [74,75]. On the other hand, more future frailty outcomes, whether negative or positive ones, are yet to be discovered.

**Figure 5:**
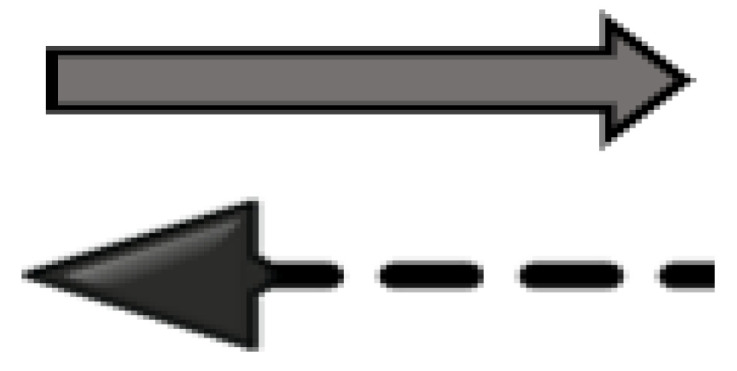
Proposed arrows between risk factors, frailty, and consequences levels.

We illustrated the prepositions between risk factors, frailty, and consequence levels in arrows (Figure 5). The solid arrows represent the risk factors leading to frailty, and frailty leading to its consequences at multiple levels as cited in the literature and as presented above. The dotted arrow represents the possible opposite direction of some frailty attributes also being antecedents/risk factors of frailty and the possible consequences of frailty leading to frailty. If true, that would support a bidirectional relationship between frailty risk factors, frailty attributes, and consequences. However, this may be true for some factors, attributes, and consequences, but not for all. For instance, older age and female gender are risk factors for frailty [34,35,36], but frailty cannot be a risk factor of aging or female gender. Nevertheless, cognitive impairment is a risk factor for frailty [34,35], an attribute of psychological frailty [23,24], and a consequence of frailty [55]. Polypharmacy is a risk factor for frailty [35], and frailty may complicate existing medical and surgical conditions; such complications may necessitate additional medications, possibly leading to polypharmacy. Thus, frailty indirectly could lead to polypharmacy. Because such bidirectional prepositions may not be inclusive to all concepts and may be indirect at times, we referred to that by dotted arrows. These dotted arrows require further research and empirical testing to confirm such bidirectional prepositions.

## 5. Limitations

Our study, proposing the mixed conceptual model of frailty, has limitations. First, the scarcity of qualitative and mixed methods studies on frailty perceptions and lived experiences in older adults might have limited our findings in this regard. Second, we relied only on a traditional literature review as a method to develop the mixed conceptual model of frailty. Other methods, such as experts’ inputs, the Delphi method, and primary data collection and analysis, could have enriched our model and added an empirical basis to it. Third, our literature review is traditional; this type of review does not follow standard reporting guidelines. Accordingly, for instance, we did not assess the risk of bias in individual studies, which could have unintentionally decreased the internal validity of our study. Fourth, initially, we utilized just two databases/academic search engines (PubMed and Google Scholar). Thus, it is possible that a more extensive search across multiple databases could have revealed more results. However, since our subject is frailty, we expect to have found the most relevant studies through PubMed.

## 6. Conclusions

We proposed a mixed conceptual model of frailty that is not meant to give a substitutable clinical definition of frailty, yielding a new tool to quantify frailty. Alternatively, the model is meant to provide a lens for clinicians and researchers to consider frailty as a concept beyond the clinical perspective and could be different from one older adult to the other, thus calling to qualify frailty in addition to quantifying it.

In research, the mixed model can serve as a base to develop further theories and research questions that can be empirically tested and qualitatively explained or explored, probably through utilizing mixed methods designs. The interpretive shift that the model emphasizes may help answer unanswered questions in frailty research. For instance, it is known that frailty is more prevalent in females [35], but it affects men more severely [76]; this paradox is not yet fully scientifically understood. The answer to such contradiction may be in understanding the lived experiences of older adults living with clinical frailty from both genders and later designing interventions based on gender differences [77]. Another question would be: How does decreased walking time affect older adults’ lives? From a clinical perspective, this could be seen as a slower time to reach a toilet, thus leading to functional incontinence. However, from the older adult perspective, this could be experienced as something else. What does it mean to live with low grip strength?

In clinical and community settings, preventing, recognizing, and managing frailty while preserving independence and autonomy and improving the quality of life of older adults is the optimal goal. This goal is best achieved through utilizing a holistic approach sensitive to what matters most to the older adult. We believe the mixed model is at the heart of such an approach. Also, the mixed model highlights the possibility that the perception and lived experience of frailty could differ by culture, geographical location, context, country’s income, available resources, living arrangements, and home health care services availability. Such differences, if they exist, would necessitate different frailty prevention, screening, and management options.

## Figures and Tables

**Table 1:** Frailty risk factors.

Frailty Risk Factors		
Socio-demographic	Increased age [34,35,36]	
	Female sex [34,35]	
	Non-Hispanic Black and Hispanic American [37]	
	African American [34]	
	Low income [34,35,36]	
	Low socioeconomic status [34]	
	Low educational level [34,35]	
	Living alone [35]	
	Being widowed or single [35]	
	Disorderly households [38]	
	Social isolation [39]	
	Poor social interaction [40]	
	Decreased social support [22]	
Biological	Inflammation	Increase in Interleukin-6 [34,41,42]
		Increase in TNF-α [34,41,42]
		Increase in C-reactive protein [34,41,42]
		Increase in fibrinogen [34,42]
		Increase in D-dimer [34,42]
		Increase in leukocytes [34,42]
		Increase in monocytes and lymphocytes [34,42]
	Hormonal Changes	Low levels of free testosterone [34,42]
		Low level of estradiol [34,42]
		Lowering in dehydroepiandrosterone sulfate (DHEAS) [34,42]
		Elevated cortisol–DHEAS ratio [34,42]
		Decreased growth hormone [34,42]
		Decreased insulin-like growth factor-1 [34,42]
	Other Biological Factors	Abnormal albumin level [34]
		Increased uric acid [34]
		Lower levels of 25-hydroxyvitamin D [34]
		Multiple deficiencies in micronutrients [34]
		Changes in glycoproteins, including HbA1c [43]
		Variability in resting metabolic rate [22]
Physical	Chronic diseases and comorbidity mainly: ▪ Cardiovascular diseases such as ischemic heart disease, heart failure, and hypertension [35,36] ▪ Chronic obstructive pulmonary disease [35,36] ▪ Type 2 diabetes mellitus [35,36] ▪ Arthritis [35,36] ▪ Anemia [44] ▪ Renal disease [45] ▪ Osteopenia [46] ▪ Stroke [35,36] ▪ Balance disorders [46] ▪ Cancer [36]	
	Malnutrition [35]	
	Being underweight, overweight, or obese [34,36]	
	Being overweight or obese in adulthood [42]	
	Sarcopenic obesity in older adults [22,42]	
	Genetic factors [42]	
	Functional limitations as per ADL and IADL [35]	
	Limitation in extremities’ function [34]	
	High allostatic load [34]	
	Urinary incontinence [35]	
	Edentulism and poor oral health [47]	
Psychological and Cognitive	Cognitive impairment [34,35]	
	Dementia [42]	
	Depression [34,35,36]	
	Spouse’s depression [34]	
	Loneliness feeling [36,39]	
	Poor self-reported health [34,35,36]	
	Low positive affect [34]	
Behavioral	Smoking [34,36]	
	Alcohol consumption [34,36]	
	Physical inactivity and sedentary lifestyle [48]	
	Low mental activity [14]	
	Irregular eating habits and low variety in food [22,49]	
	Messy body—self-presentation in the form of a disheveled appearance or poor hygiene [26]	
Situational and Environmental	Polypharmacy [35]	
	Crowded neighborhood [34]	
	Increased number of falls in the previous year [34]	
	Use of hormones [34]	
	Cold home [26]	
	Increased blood lead level [50]	
	Living in deprived areas [51]	
	Neighborhood safety [15]	
	Proximity to shops and healthcare services [15]	
	Physical/sexual/verbal abuse [52]	
	Incarceration [52]	
	Homelessness [52]	

## Data Availability

Not applicable.
